# N,Zn-Doped Fluorescent Sensor Based on Carbon Dots for the Subnanomolar Detection of Soluble Cr(VI) Ions

**DOI:** 10.3390/s23031632

**Published:** 2023-02-02

**Authors:** Enoch Kwasi Adotey, Mehdi Amouei Torkmahalleh, Philip K. Hopke, Mannix P. Balanay

**Affiliations:** 1 Department of Chemical and Material Engineering, Nazarbayev University, Astana 010000, Kazakhstan; 2 Division of Environmental and Occupational Health Sciences, School of Public Health, University of Illinois at Chicago, Chicago, IL 60612, USA; 3 Department of Public Health Sciences, University of Rochester School of Medicine and Dentistry, Rochester, NY 14642, USA; 4 Department of Chemistry, Nazarbayev University, Astana 010000, Kazakhstan

**Keywords:** carbon dots, citric acid, 4-pyridinecarboxyaldehyde, fluorescence, inner filter effect, XPS

## Abstract

The development of a fluorescent sensor has attracted much attention for the detection of various toxic pollutants in the environment. In this work, fluorescent carbon dots (N,Zn-CDs) doped with nitrogen and zinc were synthesized using citric acid monohydrate and 4-pyridinecarboxyaldehyde as carbon and nitrogen sources, respectively. The synthesized N,Zn-CDs served as an “off” fluorescence detector for the rapid and sensitive detection of hexavalent chromium ions (Cr(VI)). The zinc metal integrated into the heteroatomic fluorescent carbon dot played a functional role by creating a coordination site for the hydrogen ions that were displaced after the addition of Cr to the solution matrix. The stepwise addition of Cr(VI) effectively quenched the fluorescence intensity of the N,Zn-CDs, and this phenomenon was attributed to the internal filter effect. A low detection limit of 0.47 nmol/L for Cr(VI) was achieved in the fluorescence experiments. Real water samples were used to evaluate the practical application of N,Zn-CDs for the quantification of Cr(VI). The results show acceptable recoveries and agreement with ion chromatography-ultraviolet spectrometry results. These good recoveries indicate that the fluorescence probe is very well suited for environmental measurements.

## 1. Introduction

Measures to curb environmental pollution have necessitated the development of advanced materials that can detect and adsorb toxic substances in humans and the environment [1,2,3,4,5]. Chromium can be a hazardous substance. It occurs in the environment mainly in the trivalent (Cr(III)) and hexavalent (Cr(VI)) forms. Trivalent chromium is nontoxic and is required in the human body for the proper functioning of living tissues. However, Cr(VI) is a known human carcinogen that also has adverse health effects, such as nasal damage, asthma, bronchitis, and pneumonia [6,7,8]. To quantify the level of Cr exposure in the environment, various methods such as UV-Vis spectrophotometry [9], ion chromatography [10,11,12], atomic absorption spectrophotometry [13], and electrochemical methods [14] have been used. Although these traditional methods are widely used, they still have drawbacks, such as high cost, extensive professional operation, time-consuming operation, and unsuitability for real-time measurements [15,16,17]. Therefore, a more robust technique is needed for the rapid detection of Cr(VI) ions. 

**Scheme 1:**
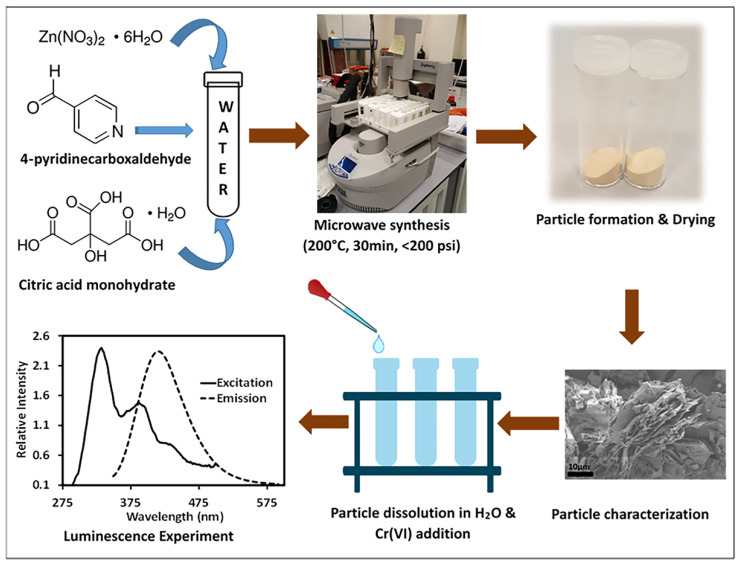
The synthesis and fluorescence experimental pathway of N,Zn-CDs.

Currently, fluorescence spectroscopy using nanomaterials for real-time monitoring is gaining popularity because it offers distinct advantages such as fast response time, specificity, and sensitive detection [18]. Carbon dots (CDs), a new fluorescent nanomaterial, have attracted attention because of their properties such as good water solubility, variable fluorescence emission, high biocompatibility, good photostability, and low toxicity [18,19,20,21]. These outstanding properties, including their chemical inertness, have made them suitable materials for photocatalysts, energy conversion, sensors, and bioimaging [1,22,23,24,25]. Various synthesis methods for fluorescent CDs have been developed, including arc discharge [26], electrochemical etching [27], laser ablation [28], hydrothermal processes [19], and ultrasonic synthesis [29]. Most of these methods often involve low quantum yield (QY), expensive equipment, and/or complicated professional handling [21]. Alternatively, microwave methods for the fabrication of CDs are gaining attention due to their ease of use, fast synthesis time, simplicity, and low cost [30]. Properties such as the electron transfer capability of CDs enhance their fluorescence properties compared to other carbon-based structures such as carbon nanotubes, carbon nanofibers, and graphene [18,31]. Currently, doping CDs with certain elements plays an important role in changing their band gap energy, which improves the charge separation efficiency of photosensors [31]. Therefore, nitrogen-doped carbon dots (N-CDs) are now an important species in the development of composite sensors. Since zinc has also been identified as insignificantly toxic, its use as a dopant in the CD matrix is beneficial for improving the physicochemical properties of carbon nanomaterials [32]. Additionally, researchers have found that the Zn dopant has relatively negligible toxicity to CDs and can therefore be considered a more environmentally friendly metal atom [32,33]. In the development of doped carbon dot sensors, guest molecules are introduced into the interior of the host carbon material. In this way, the host binds to the selective guest molecules introduced before other competing species and registers the binding event with a unique structural shape [15,16,34]. In this study, we use very simple and commercially available organic ligands (4-pyridinecarboxaldehyde and citric acid monohydrate) in the presence of Zn^2+^ ions (Scheme 1). Ligand-based fluorescence emissions are usually attributed to particles containing d_10_ metal ions that do not undergo d-d transitions. Therefore, the presence of zinc metal in the fluorescent carbon dots with mixed ligands can produce this type of ligand-based fluorescence emission [34]. Immobilized Lewis base pyridyl nitrogen sites located on the organic structure exhibit fluorescence properties and can selectively respond to metal ions [34,35]. Techniques such as microwave irradiation [32,36,37,38] and hydrothermal treatment [33] have been reported in the literature for the synthesis of N,Zn CDs, and these CDs have mostly been used as biosensors or for detecting Fe(III). However, there is limited research showing that N,Zn-CDs could serve as fluorescent sensors for detecting Cr(VI) ions.

This work aimed to synthesize doped fluorescent carbon dots (hereafter referred to as N,Zn-CDs) and to investigate their selective sensing ability for detecting Cr(VI) ions. The mechanism of Cr(VI) detection was identified with the inner filter effect (IFE) [19]. The QY of N,Zn-CDs calculated based on the absolute method [39] averaged 13.6% in this study. The carbon material showed excellent fluorescence stability after many days. Additionally, the synthesis was performed using a microwave system to demonstrate the simplicity, convenience, and short duration of the method. In most conventional methods in the literature, the aqueous solution from the synthesis is dialyzed, a rather time-consuming process to obtain the doped CD before it is used for detecting Cr(VI). However, this study used precipitates in the form of solid particles obtained directly from the synthesis process. Thus, this study is among the very few that have used solid particles as doped CD sensors for the quantification of Cr(VI).

## 2. Experimental

### 2.1. Materials and Methods

Zinc nitrate hexahydrate (Zn(NO_3_)_2_·6H_2_O, purity ≥ 98%), citric acid monohydrate (C_6_H_8_O_7_·H_2_O, purity ≥ 98%), 4-pyridinecarboxaldehyde (purity 98%), Cr(VI) standard solution (*Trace*CERT^®^, 1000 mg/L Cr(VI) in H_2_O), hydrochloric acid (ACS reagent 37%), nitric acid (ACS reagent 70%), acetone, methanol, ethanol, formamide, acetonitrile, isopropyl alcohol, and chloroform were all purchased from Sigma-Aldrich (St. Louis, MO, USA) and used without further purification. Ultrapure water was prepared using a Milli-Q water purification system with an electrical resistivity of 18.25 MΩ cm. The CEM Focused Microwave™ Synthesis System, Discover^®^ SP, was used to perform the chemical reactions under controlled conditions and synthesize the nanomaterials. Other instruments used in this study are listed in the Supplementary Materials.

### 2.2. Synthesis of N,Zn-Doped CDs

The synthesized CDs were obtained by a microwave reaction of zinc nitrate hexahydrate, citric acid monohydrate, and 4-pyridinecarboxaldehyde in H_2_O as the solvent. The process was studied by varying both the duration and ratios of the precursors (as shown in the Supplementary Materials) during the synthesis to obtain the optimum conditions for developing the fluorescent probe. Zn(NO_3_)_2_·6H_2_O (235.50 mg, 1 mmol) was measured into a 35 mL CEM microwave vessel containing 10 mL of solvent (H_2_O). Citric acid monohydrate (420.2 mg, 2 mmol) and 4-pyridinecarboxaldehyde (200 µL, 2.1 mmol) were added, and stirred for 5 min. The optimal conditions for microwave synthesis were as follows: Heating within 10 min to 200 °C with constant stirring and holding at 30 min with a pressure of less than 200 psi. The particles formed in the brown solution were separated by centrifugation and purified. The brownish precipitates were collected and dried overnight in vacuo at 70 °C. Scheme 1 illustrates the synthesis process of N,Zn-CDs and the fluorescence experiment performed. 

### 2.3. Fluorescence Titration Experiment

Three milligrams of the as-synthesized nanoparticles were dispersed in 5 mL of water and sonicated for 10 min. A 10 µg/L Cr(VI) solution was prepared from a Cr(VI) standard solution (TraceCERT^®^, 1000 mg/L Cr(VI) in H_2_O). Different volumes of the 10 µg/L Cr(VI) standard solution were added to the aqueous suspension of N,Zn-CDs, and the emission spectra were recorded. The area under the curve was determined for each peak obtained during the titration. Six replicates of the nanoparticles were analyzed, and the added Cr(VI) concentrations were plotted against the area difference (between the blank CD suspension and the obtained fluorescence intensity). The slope of the regression line was determined by statistical analysis, and the limit of detection (LOD) was calculated as follows: (3 × standard deviation/slope).

### 2.4. Fluorescence Anti-Interference Experiments

The salt solutions were prepared by dissolving known masses of the ions in 5 mL of water to obtain a 2.0 mmol/L ionic solution. The ionic solutions included *Q*-Cl_X_ (*Q* = Sn^2+^, Ca^2+^, Na^+^, Li^+^, Co^2+^, NH_4_^+^, K^+^, Zn^2+^, Fe^3+^, and Cu^2+^), MgSO_4_, Pb(NO_3_)_2_, Ni(NO_3_)_2_·6H_2_O, CrCl_3_·6H_2_O, FeSO_4_·6H_2_O, CsNO_3_, and RbNO_3_ as cations, and Na_x_-*R* (*R* = NO_2_^−^, CO_3_^2−^, HCO_3_^−^, SO_4_^2−^, OH^−^, PO_4_^3−^) and K_x_-*T* (*T* = Br^−^, I, IO_3_^−^, NO_3_^−^, MnO_4_^−^) as the anions. Then, 10 µL of the ion solutions were added to the N,Zn-CDs solution (3 mg/5 mL), and the fluorescence intensity was measured at an excitation wavelength of 330 nm. Finally, an equivalent amount, 500 µL of a 10 µg/L Cr(VI) standard solution, was added to 2 mL of the ionic solutions, and the corresponding fluorescence intensity was recorded.

### 2.5. pH and Solvent Stability 

N,Zn-CDs (25 mg) were dispersed in 30 mL of ultrapure water and sonicated for 10 min. Of this, 2 mL was added to centrifuge tubes containing 5 mL of ultrapure water in the pH range of 2.0 to 13.0 (pH was adjusted with 0.1 mol/L HCl and/or NaOH). After 5 min of equilibration time in the cuvette, the fluorescence spectra were recorded. About 3.0 mg of N,Zn-CDs was added to 5.0 mL of various solvents (DMF, formamide, acetonitrile, isopropyl alcohol, acetone, methanol, ethanol, and chloroform) at room temperature to test their fluorescence stability.

### 2.6. UV-Vis Absorption Experiment

The UV-Vis absorption spectra were recorded using the Evolution 300 UV-Vis spectrophotometer with dual-beam optics, which uses a xenon flash lamp as the light source for the spectral range of 190–1100 nm. The absorption spectra were recorded after transferring the fine suspensions of N,Zn-CDs (3 mg/5 mL) and 50 µL of a 10 µg/L Cr(VI) standard solution into a quartz cuvette.

### 2.7. Zeta Potential Titration Experiment

Similar to the fluorescence titration experiment, a suspension of N,Zn-CDs (3 mg/5 mL) and Cr(VI) standard solution was used to study the electrostatic interaction between the nanomaterial and Cr(VI). Zeta potential titration was performed by adding 50 µL of 10 µg/L Cr(VI) standard solution stepwise to the aqueous suspension of N,Zn-CDs. The potentials of the particles were measured three times to obtain an average value.

## 3. Results and Discussion

### 3.1. Characterization of the (N,Zn)-Doped Fluorescent CDs

**Figure 1:**
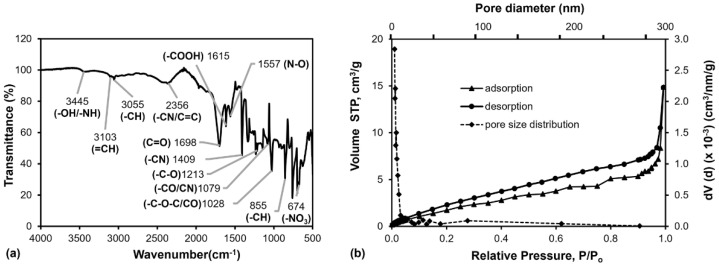
(**a**) FTIR spectra and (**b**) N_2_ adsorption-desorption isotherms with the corresponding pore size distribution of the as-synthesized nanomaterial.

A small peak at 3444 cm^−1^ was observed in the FTIR spectrum (Figure 1a), which can be attributed to the stretching vibration of the -OH group in the structure of the nanomaterial [19,20,26,40,41,42]. The weakly pronounced peaks at 3103 and 3054 cm^−1^ can be attributed to the vibrational bands of =CH and -CH, respectively [19,41,42]. The -CN peaks [26,40,41] at 2356, 1408, and 1079 cm^−1^ are the stretching vibrations of the pyridyl nitrogen, indicating that 4-pyridinecarboxaldehyde was fully involved in the formation of the nanomaterial. The peaks at 1697, 1213, 1079, and 1027 cm^−1^ are assigned to the C=O stretching vibrations of the aromatic rings in 4-pyridinecarboxaldehyde and the carboxyl group in citric acid [34]. The peak at 1339 cm^−1^ is attributed to the -CHO out-of-plane deformation and the C-N stretching vibration of 4-pyridinecarboxyaldehyde [34,43]. The vibrational band at 1615 cm^−1^ is attributed to the asymmetric stretching vibration of the carboxyl functional group (-COOH) in citric acid [20,43,44]. A peak at 759 cm^−1^ could be attributed to the in-plane and out-of-plane stretching of the aromatic C-H groups of benzene rings [43,44]. The incorporation of nitrates usually leads to typical peaks at 1380, 839, and 670 cm^−1^, and in our study, the visible peak at 673 cm^−1^ can be attributed to the antisymmetric deformation of NO_3_^−^ from the zinc nitrate incorporated in the synthesis. 

N_2_ adsorption-desorption isotherms (Figure 1b) were performed to determine the porosity and Brunauer-Emmett-Teller surface area (BET) of the nanomaterial. The porosity of the particles was determined by measuring the nitrogen gas desorption isotherms at 77.35 K. Before gas sorption, the particles were degassed at 115 °C under vacuum. The adsorption-desorption curve showed a type IV hysteresis loop, indicating that the amount of adsorbed N_2_ was approaching the threshold value [45]. The BET surface area of the nanomaterial was determined to be 9.615 m^2^/g, comparable to some reported work [34], and had a volume of 0.012 cm^3^/g. Considering these low values, it is reasonable to assume that the nanomaterials interpenetrate, resulting in their dense packaging. This interpenetration will be discussed after studies to refine the crystalline structure of the nanomaterial. The nanoparticles were identified as mesoporous materials with a type IV adsorption behavior [45]. The pore size distribution diagram (Figure 1b) was also constructed from the adsorption lines of the samples with a dominant pore diameter of 3.062 nm.

**Figure 2:**
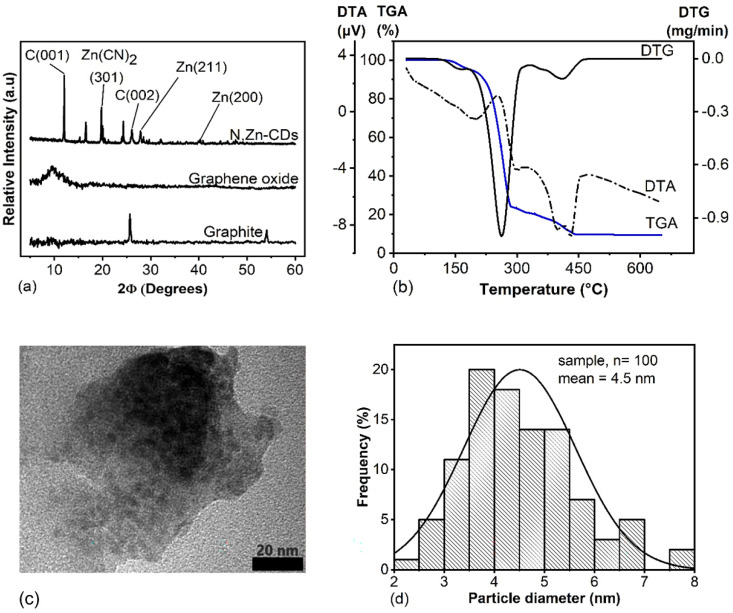
(**a**) XRD patterns for graphite and graphene oxide compared to the as-synthesized N,Zn-CDs, (**b**) TGA, DTA, and DTG patterns, (**c**) TEM image, and (**d**) particle size distribution of the as-synthesized N,Zn-CDs.

The powder XRD patterns of the (N,Zn)-doped fluorescent carbon dots were characterized to show their crystalline nature. As shown in Figure 2a, there were distinct diffraction peaks in the patterns at 12.04°, 16.52°, 19.75°, 24.32°, 26.12°, and 27.91°, respectively. The corresponding interlayer spacing (d) was calculated using Bragg’s law (the Cu-Kα (λ) wavelength is 0.154 nm). The high diffraction peak around 12.04° (d = 7.35 Å) appears similar to the typical characteristic peak of graphene oxide (2θ = 10.6°, {001} plane) [46], possibly due to an increase in the sp^3^ interlayer distance during synthesis. This value differs slightly from that of the original graphene oxide because the functional groups such as aldehyde, hydroxyl, and carbonyl groups were incorporated into the nanomaterial during the synthesis process and are bound to the edges of the basal planes of the crystal structure. The diffraction peak at 19.75° (d = 4.49 Å) is identified with the phase name zinc cyanide {301} and 24.32° (d = 3.66 Å) as the carbon phase. The diffraction peak at 26.12° (d = 3.41 Å) resembles the characteristic peak of graphite ({002} planes, 2θ = 26.5°) [47]. This close resemblance to the crystal structure of graphite could be due to the highly ordered carbon phase in which the sp^2^ layer (C-C) spacing did not change during the carbonization process. Other smaller diffraction peaks were observed at 2θ values of 27.91°, 32.02°, 40.26°, and 47.25°, which were associated with Zn phases in the nanomaterial with mixed ligands. 

The TGA, DTG, and DTA curves of N,Zn-CDs are shown in Figure 2b. For the TGA, the excess solvent molecules were removed from the pores and then thermally activated [16]. The results show that the N,Zn-CDs release their internal free and terminal solvent over the temperature range from 100 °C to 480 °C. The DTG curve shows four stages of decomposition. The short plateau visible in the 1st stage, about 4.6% in the range from 100.2 °C to 180.4 °C, was attributed to the release of free H_2_O and unreacted 4-pyridinecarboxyaldehyde from the pores of the particles. The second weight loss from 180.4 to 330.9 °C (74.8%) was due to the removal of possible phenyl rings from the ligand (4-pyridinecarboxyaldehyde). Further on, the N,Zn-CDs decomposed in two stages due to the probable decomposition of the nitrate precursor and the release of nitrogen oxides at 330.9–372.4 °C (2.8%) and at 372.4–478.3 °C (8.3%). The DTA curve shows a broad endothermic peak at 193.4 °C, corresponding to the first stage of decomposition. An exothermic peak observed at 254.6 °C and a small peak at 321.3 °C correspond to the second stage of decomposition. Two endothermic peaks observed at 396.6 °C and 430.4 °C correspond to the third and fourth stages of decomposition in the DTG curve, respectively. Thus, the results of TGA, DTG, and DTA show the thermal decomposition pattern of N,Zn-CDs.

The TEM image and the size distribution of the N,Zn CDs are shown in Figure 2c,d, respectively. The images show that the N,Zn-CDs are monodispersed in solution and have a nearly spherical shape. Analysis of the N,Zn CDs using ImageJ software revealed an average particle diameter of 4.5 nm, after measuring 100 particles from the TEM images. The results of the EDS spectrum (Figure S1a) show that the elements carbon (C), oxygen (O), nitrogen (N), and zinc (Zn) are strongly represented with 61.0%, 24.2%, 14.1%, and 0.8%, respectively. Thus, the quantitative analysis of EDS confirmed the inclusion of nitrogen, zinc, and carbon in the structure of the nanomaterial. The SEM analysis and elemental mapping performed on the solid particles are shown in Figure S1 in the Supplementary Materials. 

### 3.2. Understanding Cr(VI)-Organic Matrix Mechanism Using XPS

**Figure 3:**
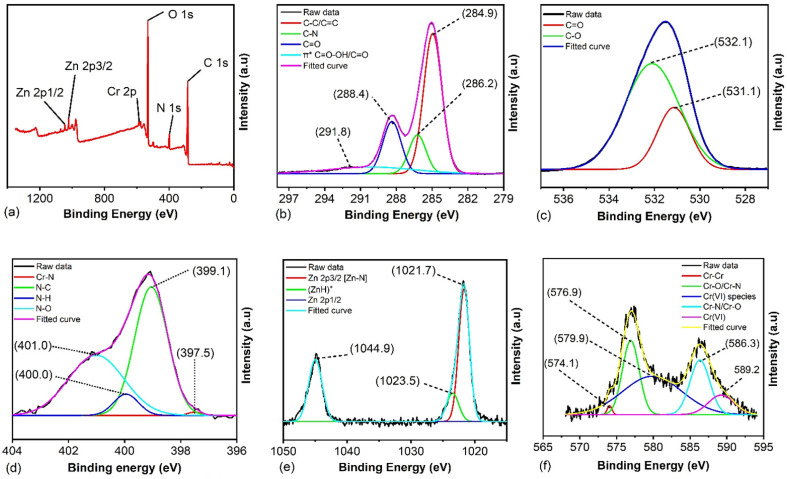
XPS spectra of N,Zn-CDs + Cr(VI) system (**a**) survey spectrum, (**b**) carbon deconvoluted peaks (C 1s), (**c**) oxygen deconvoluted peaks (O 1s), (**d**) nitrogen deconvoluted peaks (N 1s), (**e**) zinc deconvoluted peaks (Zn 2p), and (**f**) chromium deconvoluted peaks. * The mixed Cr(VI) species identified within 579 eV are those of Cr(VI)/CrO_3_, Cr(VI)/CrO_4_^2−^, and Cr(VI)/Cr_2_O_7_^2−^ [52].

**Figure 4:**
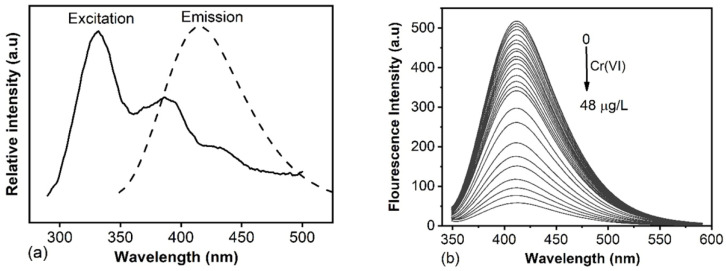
(**a**) Excitation and emission wavelengths of the synthesized particles and (**b**) fluorescence quenching spectra of N,Zn-doped fluorescent carbon dots as a function of different concentrations of Cr(VI). Excitation was performed at 330 nm.

XPS analysis was performed to identify the chemical constituents of the nanomaterial. The spectrum in Figure S2a shows the presence of C, N, O, and Zn elements in the particles, confirming the doping of the carbonaceous material with N, O, and Zn groups. The estimated atomic C:O:N:Zn ratios of 60.3:32.0:6.9:0.8 show the enriched N, O, and Zn functionalities within the carbon motif, which ensure the high hydrophilicity of the material in an aqueous medium. The chemical bonding information was investigated by deconvolution and fitting of the C1s, N1s, Zn2p, and O1s spectra. Figure S2b shows that the C1s spectrum mainly contains C-C/C=C (284.3 eV), C-N (285.7 eV), and C=O (288.0 eV) groups. The detailed O1s spectra in Figure S2c show the presence of O-H (529.5 eV), C=O (531.0 eV), and C-O (532.8 eV) groups within the carbon motif [48]. In addition, the N1s spectrum in Figure S2d was fitted to show the presence of N-Zn (398.8 eV), N-H (400.5 eV), and N-O (401.3 eV) bonds [20,48], confirming the successful incorporation of nitrogen into the nanomaterial. The deconvolution of Zn2p in Figure S2e also reveals the presence of Zn2p1/2 (1044.3 eV) and Zn2p3/2 (1021.2 eV, Zn-N) in the nanomaterial [32]. These XPS results are further evidence that abundant N, Zn, and O groups were incorporated in/on the nanomaterial. These groups not only increase the hydrophilicity of the material but also contribute to the chromophoric/auxochromic properties of the fluorescence spectra [48]. In addition, the N,Zn-CDs were exposed to a 1000 ppm Cr(VI) solution to study the interaction between the Cr(VI) and other elements in the organic matrix (C, O, N, and Zn) by XPS (see Figure 3). This high concentration of the Cr(VI) was used to obtain a good signal-to-noise ratio during XPS analysis and to have protonated pyridyl, aldehyde, and hydroxyl groups in the composite. The nanomaterials were hydrophilic, and the resulting solution had a pH of ~6.8 after the addition of Cr(VI), which could prevent the conversion between Cr(VI) and Cr(III) as previously described in the context of the chemistry of Cr(VI) [49]. The mechanism of Cr(VI)-N,Zn-CDs matrix binding was investigated using the detailed XPS spectra before (Figure S2) and after (Figure 3) the addition of Cr(VI) to the solution. We found that the N,Zn-CDs were already water soluble, so it was easy to determine the variability after successful incorporation of Cr(VI) to determine the elemental binding that might occur in the system. The XPS spectrum in Figure 3a shows the presence of C, N, O, Zn, and Cr in the particles after the addition of the Cr(VI) standard solution into the carbonaceous material. Comparison of the deconvoluted C1s peaks in Figure 3b with Figure S2b shows the presence of an additional carbon peak at 291.8 eV, that was not originally present in Figure S2b. Although we were unable to determine the exact assignment of the 291.8 eV feature on C1s, we suspect that it may be related to the π* C=O-OH/C=O groups (π-π* shake up satellite) [50,51]. This phenomenon is related to the possible oxidation of the graphite lattice (oxidized states of carbon and perhaps other functional groups) [50,51] in the N,Zn-CDs. The C-N peak indicates that free nitrogen is still present after the introduction of Cr(VI) species. However, the C=O (288.4 eV), C-N (286.2 eV), and C-C/C=C (284.9 eV) carbon groups are still present, although they are red-shifted after the addition of Cr (Figure 4b). Figure 3c shows the disappearance of the O-H groups in the deconvoluted O1s peaks originally seen in Figure S2c (Supplementary Materials). 

However, the C-O peak in Figure 3c was enhanced while the intensity of the C=O peak decreased, a phenomenon that is reversed in Figure S2c (Supplementary Materials). The disappearance of O-H in Figure 3c indicates the presence of free O^2−^/H^+^ species in solution and shows enhanced N-O (401.0 eV) in the deconvoluted peaks of N1s (Figure 3d), compared to the original peak in Figure S2c (Supplementary Materials). The binding of Cr to the nitrogen moiety was observed with the Cr-N group at 397.5 eV. The presence of free O^2−^/H^+^ species in the solution led to the formation of a small peak of (ZnH)^+^ species (1023.5 eV) [53] as shown in Figure 3e. Thus, the zinc metal present in this nanomaterial played a functional role by creating a coordination site for the hydrogen ions that were displaced after the addition of Cr in the matrix. Figure 3f shows the deconvoluted XPS spectra of Cr2p after the addition of Cr(VI) to the nanomaterial.

The deconvolution results of the Cr2p3/2 peak showed two broad peaks at 579.9 eV and 576.9 eV and a small peak at 574.1 eV, respectively, assigned to Cr(VI)-mixed species, Cr-O/Cr-N, and elemental Cr (Cr-Cr) [52,54]. Generally, the Cr(VI)-mixed species identified at 579 eV are Cr(VI)/CrO_3_, Cr(VI)/CrO_4_^2−^, and Cr(VI)/Cr_2_O_7_^2−^ [52]. Similarly, the binding energies of the Cr2p1/2 peaks at 586.3 eV and 589.2 eV correspond to Cr-N/Cr-O states and Cr(VI)-mixed species, respectively [54].

### 3.3. Fluorescence Experiment of N,Zn-CDs on Cr(VI) Detection

The excitation and emission spectra of the fluorescent carbon dots with mixed ligands were measured at room temperature and are shown in Figure 4a. The excitation peak at 330 nm was attributed to ligand-centered transitions, while the peaks at 385 and 432 nm were generally identified as metal-to-ligand charge transfer excitations. The latter two excitation peaks did not yield spectra upon emission when combined with Cr(VI). Therefore, the excitation peak of 330 nm emitting at 417 nm was chosen for all experiments. Ligand-based fluorescence emissions are attributed to particles containing metal ions without d-d transitions [34]. The presence of zinc metal in the fluorescent carbon dots with mixed ligands had the potential to produce this type of ligand-based fluorescence emission. Meanwhile, the stability of the solution after the addition of Cr(VI) was also investigated, as shown in Figure S2f. No significant change in the quenching stability of the nanomaterial suspension was observed even after seven minutes. Therefore, a maximum waiting time of 1 min was used in all experiments before the measurement was performed on the fluorescence spectrometer.

To investigate the ability of the nanomaterial to detect Cr(VI) ions, an aqueous solution of the particles was prepared, and Cr(VI) standard solutions were gradually added to the suspension. Figure 4b shows the quenching effect upon the addition of different concentrations of Cr(VI). This effect could be due to the interaction of the chromium ions with the pyridyl-nitrogen binding sites in the framework of the nanomaterial when it is in solution. Previous work by Chang Liu and Bing Yan [16], who used imidazolate-2-carboxaldehyde to synthesize a zeolitic imidazole framework, showed that both nitrogen sites and uncoordinated aldehyde groups conferred selectivity for metal ions to their MOF. In this regard, the mechanism of fluorescence quenching of the presently synthesized nanomaterial and Cr(VI) ions could be attributed to the presence of weak atomic interactions of the available pyridyl nitrogen atoms as well as to the coordination of the aldehyde group located on the 4-pyridinecarboxaldehyde and its interaction with Cr(VI) ions [16,35]. Our previous studies with 3-pyridinecarboxaldehyde [34] also explained that the weak binding between the pyridyl nitrogen and the aldehyde in the ligand contributes to a decrease in the intraligand fluorescence efficiency with increasing chromate/dichromate concentration. A typical interaction (N---CO) in the complexes of pyridine with carbonyl compounds is the weak n-π* interaction, resulting from the delocalization of the lone pair (n) of pyridyl nitrogen atoms into the antibonding (π*) orbital of the available aldehyde (a carbonyl group) [55,56].

For the calibration curve, the area difference under the curve (between the blank N,Zn CDs and the subsequent curve obtained after the addition of Cr(VI)) was plotted against the concentration of Cr(VI) ions. Figure S3c (Supplementary Materials) shows a narrow linear trend when the Cr(VI) concentration is low and logarithmic trend when the concentration is high. However, in this study, the linear trend (Figure S3c, Supplementary Materials) was considered with a regression coefficient of R^2^ = 0.9991, and the equation of the line is y = 5013.9x + 184.25. The calculated detection limit of 24.26 ng/L (0.47 nmol/L) Cr(VI) ions was determined based on the 3δ/k relationship (δ represents the standard deviation of 6 replicates, k is the slope of the calibration curve). Table 1 shows the performance of the synthesized N,Zn CDs compared to already known CDs used for the detection of Cr(VI) ions in the aqueous phase. Our proposed N,Zn-CDs show higher sensitivity compared to previous sensors, albeit over a narrow range, for the detection of Cr (VI). Based on the use of nitrogen and zinc co-doped carbon dots in the synthesis of fluorescent probes for the detection of Cr(VI), our LOD (0.47 nmol/L) shows the second lowest LOD after the work of Wang et al. [57], who synthesized a nitrogen-sulfur-doped multifunctional fluorescent carbon dot (N,S-CDs) for the detection of Cr(VI) and Ba(II). However, their work was carried out over a longer synthesis period. In contrast, our study was performed within 30 min synthesis time using simple ligands to achieve a competitive LOD that is lower than most of the reported LODs.

### 3.4. Effect of pH

The speciation of Cr(VI) depends on the pH and their concentrations [49]. Since the N,Zn-CDs matrix has a pH of ~4.5, we expect HCrO_4_^−^ and Cr_2_O_7_^2−^ to be the dominant Cr species present in solution during the measurement. The Cr(VI) species used in this study was in the form of NH_4_(CrO_4_). During the reversible reaction with chromate (CrO_4_^2−^) ions and H^+^, Cr_2_O_7_^2−^ ions are ionized in aqueous solution according to Equation (1) [58]:

2CrO_4_^2−^ + 2H^+^ ↔ Cr_2_O_7_^2−^ + H_2_O (1)

Therefore, we expect Cr (VI) ions in solution to interact with the N,Zn-CDs via the pyridyl nitrogen (-NH), hydroxyl (-OH), and aldehyde (-CHO) groups. Similar to the work of Bandara et al. [58], the pyridyl nitrogen in the N,Zn-CDs will typically follow Equation (2) at these acidic pH values:

R − NH + H^+^ → R − NH_2_^+^ (2)

This equation implies that the pyridyl groups in the nanomaterial remain positively charged. Therefore, we can theorize that the pyridyl nitrogen is responsible for coordinating the Cr(VI) species from the aqueous solution by electron transfer with the negatively charged Cr(VI) ions to cause the quenching of the fluorescent N,Zn CDs.

**Figure 5:**
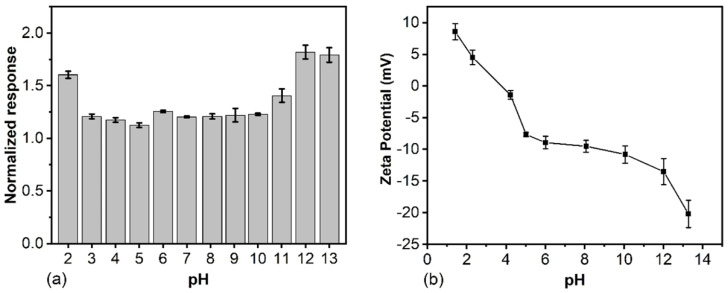
(**a**) The effect of varying pH on the fluorescent intensity and (**b**) the zeta potential with varying pH of the nanomaterial in a suspension of DI water.

The effect of pH on the fluorescence of the nanomaterial was investigated by adding a negligible amount of 0.1M HCl and/or NaOH to the suspension. Figure 5a shows the effect of pH on the fluorescence intensity of the suspension. The pH ~4.5 was measured in our study when we dissolved the N,Zn-CDs in water and was also used in all other experiments. The solution pH is an important parameter for the sorption process of a sensor because it can affect the surface chemistry of the adsorbent and the species of the impurities of interest [59]. As shown in Figure 5a, the fluorescence intensity of the nanomaterial did not change significantly from ~pH 3 to 10, indicating that the particles were stable in these ranges. However, an increase in fluorescence intensity was observed at pH < 3 or pH > 10. At lower pH values, the surface charge of nanomaterials containing metals is usually positively charged, resulting in H^+^ ions competing with the available metal ions and subsequently reducing the adsorbed metal [60,61,62]. On the other hand, at higher pH values, the number of anions increases, which allows the exchange of ions by a displacement reaction with -OH, producing NaCl [61]. The same phenomenon is observed when measuring the zeta potential at different pH values, as shown in Figure 5b. At a lower pH, the surface zeta potential of the particles assumes a positive charge, while an increase in pH results in a negative surface zeta potential charge of the particles. Thus, these experiments in aqueous medium show that the nanomaterial has good stability and can function effectively in the pH range of 3–10. 

### 3.5. Mechanism of Cr(VI) Detection

**Figure 6:**
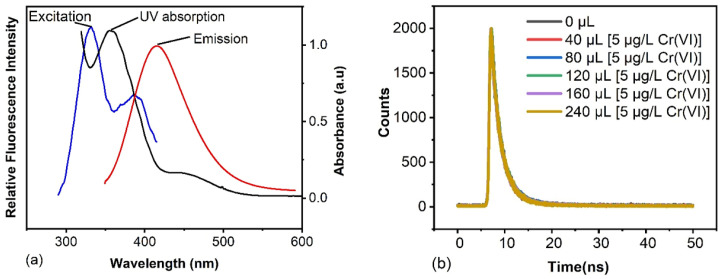
(**a**) UV-Vis absorption spectra of Cr(VI), fluorescence excitation spectra, and emission spectra of the N,Zn-doped fluorescent carbon dots in aqueous solution and (**b**) time-resolved decay of the N,Zn-CDs system at different volumes of 5 µg/L Cr(VI) concentrations.

Possible mechanisms to explain the fluorescence quenching of Cr(VI) have been associated with factors such as the inner filter effect (IFE), fluorescence resonance energy transfer (FRET), and conversion of fluorescent materials to nonfluorescent materials [63]. Given the high chemical stability we have achieved within pH 3–10 in Figure 5a, the phenomenon of conversion of fluorescent materials to nonfluorescent types is excluded for evaluation. The remaining possibilities, IFE and FRET, can be investigated by analyzing the overlap of the UV-Vis spectra when Cr(VI) is added to the nanomaterial. Figure 6a shows the overlap of the UV-Vis adsorption spectra with the excitation and emission spectra of the aqueous N,Zn- CD system. This overlap suggests that IFE may be a factor that enhances the fluorescence quenching of Cr(VI). This mechanism is that the overlap causes the chromium ions to compete with the organic ligand for the absorption of light energy [62], so that the amount of light absorbed by the ligand decreases, leading to a decrease in the efficiency of electron transfer from the ligand to the metal ions in solution. The optical properties of the nanomaterial are revealed in the UV-Vis absorption spectrum (Figure S3a, Supplementary Materials). It exhibits two characteristic absorption peaks at 299 nm and 355 nm. The strong peak at 299 nm corresponds to the n-π* transitions that typically lead to an intermolecular charge transfer from the C=O lone pair to the antibonding π* orbital of the carbonyl group. Similarly, the peak at 355 nm can be interpreted as the n-π* transition of double bonds with C=O groups [34].

To further verify the IFE mechanism in the N,Zn-CD with Cr(VI), we performed time-resolved fluorescence decay measurements on 2 mL (3 mg/5 mL N,Zn-CDs) solutions by adding different volumes (0, 40, 80, 120, 160, 240 µL) of 5 µg/L Cr(VI) concentrations. In these experiments, we obtained the fluorescence decay spectra of N,Zn-CDs in the absence and presence of Cr(VI). The recorded fluorescence lifetime values (Figure 6b) showed little difference between the replicate samples. Table S1 (Supplementary Materials) shows the fluorescence decay of the N,Zn-CD system fitted with a three-exponential function, yielding an average lifetime of ~2.6 ns. Previous studies reported that electronic energy transfer to Cr(VI) generally adds another decay channel to the excited states of N-doped carbon dots and leads to shorter lifetimes [64]. However, given the unchanged time-resolved decay process in our study (Figure 6b), it can be confirmed that the fluorescence quenching of the N,Zn-CDs suspension is controlled by the IFE of Cr(VI) (through a static quenching mechanism) and not by an energy transfer within the CD-Cr(VI) system.

### 3.6. Anti-Interference Study 

**Figure 7:**
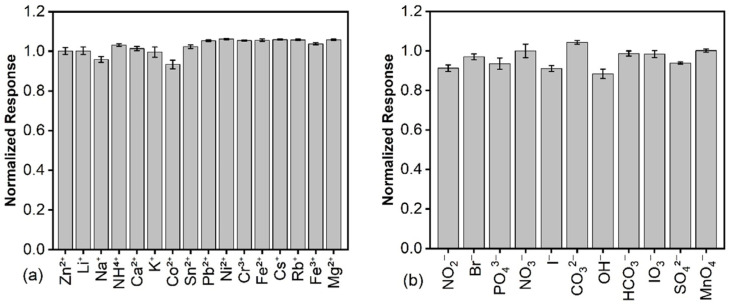
Fluorescence response of mixed-ligand fluorescent carbon dots to different (**a**) cations and (**b**) anions mixed with Cr(VI) at an excitation wavelength of 330 nm.

The quality and stability of the fluorescence of the (N,Zn)-doped fluorescent carbon dots was investigated by examining the potential interference that could occur from other ions. The solutions containing metal cations (Figure 7a) showed no significant differences in fluorescence intensity upon addition of Cr(VI) to the aqueous solution, a phenomenon consistent with previous work [34]. Thus, we conclude that the free pyridyl nitrogen atoms maintained their strong binding to the ions upon addition of cations, so the ions could not reduce the efficiency of the linkers in coordinating with the transition metal (Zn in this case), and therefore, no significant quenching of fluorescence occurred. 

The presence of possible hydroxyl groups and free carboxylic oxygen atoms in the aqueous form of the sample could also affect the interactions between the particles and anions [62]. Because this interaction could affect the emission spectra through hydrogen bonding, an evaluation of the anti-interference capability of the nanomaterial in the presence of anions was also performed. The interference by anions was also observed and investigated for Na_x_A (A = NO_2_^−^, CO_3_^2−^, HCO_3_^−^, SO_4_^2−^, OH^−^, PO_4_^3−^) and K_x_N (N = Br^−^, I, IO_3_^−^, NO_3_^−^). The result in Figure 7b shows that the anions also had a negligible effect on the fluorescence intensity of the nanomaterial in its aqueous form. This anti-interference analysis shows that the N,Zn-CDs system is able to detect Cr(VI) ions even in the midst of other competing ions, indicating that these ions are only weakly bound to the pyridyl sites of the particles in solution, which cannot cause significant interference. The significance of this conclusion implies that the synthesized N,Zn-CDs can serve as a useful fluorescent sensor for the detection of Cr(VI) in aqueous medium, even in the presence of other available ions.

The stability of the nanomaterial in different solvents was further investigated. Figure S3b shows the different fluorescence intensity depending on the type of solvent molecules used. Species such as isopropyl alcohol (IPA), methanol, and ethanol exhibit significant fluorescence-enhancing effects compared to water. Other solvents such as DMF and water appear to have similar fluorescence intensities. However, significantly lower intensities were registered upon addition of the other solvents (Figure S3b). Thus, the results provide insight into the extent of stability of our nanomaterial in different organic molecules. Considering the increased intensities registered for IPA, ethanol, and methanol, future studies could be directed towards using our nanomaterial to detect these solvents.

In addition, the stability of the N,Zn-CDs-water system was evaluated by observing its fluorescence intensity over several days (Figure S3d). The fluorescence spectra were measured at room temperature, and the measurements were performed after several days of storage. Figure S3d shows that the fluorescence intensity remained at about 94.5% of the original intensity, on average, even when stored for more than one month. This indicates that the aqueous N,Zn-CDs system achieves excellent stability even after many days of preparation.

### 3.7. Real Sample Analysis

The fluorescence sensor was used with tap water and with seawater to test the applicability of our proposed method. The tap water came from one of our laboratories and was not pretreated. The seawater samples (pH ~8.0) were obtained from the Aral Sea (45° N, 60° E), and pretreatment was performed by sampling with a BD syringe and filtering through a 0.22 µm filter (Thermo Scientific 25 mm Nalgene syringe filter SFCA) to remove particulate matter before analysis. The results of the analyses are shown in Table 2. The recoveries of the spiked samples were 98.4–104.1% and 94.2–136.24% for the tap water and seawater samples, respectively. In general, the relative standard deviation (RSD) for the doped samples was less than 5%. Thus, the fluorescent N,Zn-CDs were found to be suitable probes for the detection of Cr(VI) in an aqueous medium.

## 4. Conclusions

Nitrogen from 4-pyridinecarboxaldehyde and zinc as a dopant for the carbon dots (N,Zn-CDs) yielded a sensitive fluorescent probe for the detection of soluble Cr(VI) ions. The fluorescence intensity of the sensor decreased proportionally with an increase in the concentration of Cr(VI) anions. The anti-interference capability of the sensor in the presence of other metal ions demonstrated the sensitivity and selectivity of the probe for chromium ions. XPS characterization of the particles showed that the incorporation of Zn metal into the as-synthesized material played a functional role in creating a coordination site for the binding of the displaced H^+^ ions in the solution matrix. The N,Zn-CDs probe showed a detection limit of 0.47 nmol/L for soluble Cr(VI) species. Quantification of Cr(VI) in the environment is an emerging area of research, and given the applicability of these carbon materials at very low LOD, this nanomaterial may be a suitable material to be used as highly sensitive fluorescent probes for measuring Cr(VI) in the environment in the future.

## Figures and Tables

**Table 1:** Comparison of the as-synthesized N,Zn-CDs with some reported CD fluorescent sensors for detecting Cr(VI) ions.

Detection Probe	Synthesis Route	Linear Range (μM)	LOD (nM)	Reference
N-doped CDs	One-step hydrothermal method	0.1–430	1.26	[19]
N-doped CDs	One-step pyrolysis process	0.01–250	5	[21]
N, S co-doped CDs	Hydrothermal reaction	0.01–6.61	0.33	[57]
S, N-CDs	Acid-base neutralization and exothermic carbonization method	0.065–198	0.56	[41]
N,Zn-doped CDs	Microwave-assisted synthesis	0.005–0.135	0.47	This work

**Table 2:** Quantification of Cr(VI) in real water samples using as-synthesized N,Zn-CDs sensor or IC-UV.

Samples	Spiked (µg/L)	Found (µg/L)	Recovery (%)	RSD (%) (n = 3)	IC-UV Cr(VI) (µg/L)
Tap water	0.05	0.05	101.03	3.30	0.08
	0.10	0.10	98.37	7.39	0.15
	0.30	0.31	101.75	1.04	0.46
	0.50	0.52	104.11	1.04	0.76
Seawater	0.05	0.05	94.19	4.29	0.15
	0.10	0.10	101.99	1.32	0.22
	0.30	0.38	127.94	1.07	0.49
	0.50	0.68	136.24	0.11	0.77

RSD: relative standard deviation.

## Data Availability

Not applicable.

## Supplementary Materials

The following supporting information can be downloaded at: https://www.mdpi.com/article/10.3390/s23031632/s1, Figure S1: (a) EDS composition, (b) SEM image, and (c) to (d) elemental mapping of the as-synthesized particles; Figure S2: XPS spectra of N, Zn-CDs (a) survey spectrum, (b) carbon deconvoluted peaks (C1s), (c) oxygen deconvoluted peaks (O1s), (d) nitrogen deconvoluted peaks (N1s), and (e) zinc deconvoluted peaks (Zn2p). (e) Comparison of the quenching stability of the N,Zn-CDs suspension over a few minutes; Figure S3: (a) UV-Vis absorption spectrum of the N,Zn-CDs + Cr system; the inset shows the solution under the UV lamp at 254, 302, and 365 nm, (b) fluorescence response of the as-synthesized nano-material to various organic solvents, (c) a plot of low and high Cr(VI) concentrations when added to the N,Zn-CDs suspension (inset: the calibration curve showing the linear trend of Cr(VI) at very low concentrations), and (d) Influence on the stability of fluorescence intensity of N,Zn-CDs in water after several days; Figure S4: CD suspensions (a) at room temperature and (b) observed under a UV lamp at 365 nm; Figure S5: FTIR spectra for the variation of the precursors; Figure S6: XRD diffraction spectra for graphite and the variation of precursors; Figure S7: (a) Excitation and (b) emission spectra of the various precursor combinations; Figure S8: SEM/EDS analyses for the variation of precursors with ID (a) Z_ii_, (b) Z_iii_, (c) N_ii_, and (d) N_iii_; Table S1: The fluorescence decay of the N,Zn-CD + incremental volumes addition of 5 ppb Cr(VI) system fitted to a three-exponential function; Table S2: Variations in the use of starting materials during synthesis and its LOD.

## References

[1] Evans J.D., Garai B., Reinsch H., Li W.J., Dissegna S., Bon V., Senkovska I., Fischer R.A., Kaskel S., Janiak C. (2019). Metal-organic frameworks in Germany: From synthesis to function. Coord. Chem. Rev..

[2] Vaseashta A., Vaclavikova M., Vaseashta S., Gallios G., Roy P., Pummakarnchana O. (2007). Nanostructures in environmental pollution detection, monitoring, and remediation. Sci. Technol. Adv. Mater..

[3] Ravichandiran P., Boguszewska-Czubara A., Maslyk M., Bella A.P., Subramaniyan S.A., Johnson P.M., Shim K.S., Kim H.G., Yoe D.J. (2019). Naphthoquinone-Based Colorimetric and Fluorometric Dual-Channel Chemosensor for the Detection of Fe^2+^ Ion and its Application in Bio-Imaging of Live Cells and Zebrafish. ACS Sustain. Chem. Eng..

[4] Ravichandiran P., Subramaniyan S.A., Bella A.P., Johnson P.M., Kim A.R., Shim K.S., Yoo D.J. (2019). Simple Fluorescence Turn-On Chemosensor for Selective Detection of Ba^2+^ Ion and its Live Cell Imaging. Anal. Chem..

[5] Ravichandiran P., Boguszewska-Czubara A., Maslyk M., Bella A.P., Johnson P.M., Subramaniyan S.A., Shim K.S., Yoo D.J. (2020). A phenoxazine-based fluorescent chemosensor for dual channel detection of Cd^2+^ and CN^−^ ions and its application to bio-imaging in live cells and zebrafish. Dyes Pigments.

[6] Leonard A., Lauwerys R.R. (1980). Carcinogenicity and mutagenicity of chromium. Mutat. Res..

[7] Huvinen M., Uitti J., Oksa P., Palmroos P., Laippala P. (2002). Respiratory health effects of long-term exposure to different chromium species in stainless steel production. Occup. Med..

[8] Norseth T. (1981). The carcinogenicity of chromium. Environ. Health Perspect..

[9] Fournier-Salaün M.-C., Salaün P. (2007). Quantitative determination of hexavalent chromium in aqueous solutions by UV-Vis spectrophotometer. Cent. Eur. J. Chem..

[10] Wojasińska E., Trojanowicz M. (1996). Ion chromatographic speciation of chromium with diphenylcarbazide-based spectrophotometric detection. J. Chromatogr. A.

[11] Inoue Y., Sakai T., Kumagai H. (1995). Simultaneous Determination of Chromium(III) and Chromium(VI) by Ion Chromatography with Inductively-Coupled Plasma-Mass Spectrometry. J. Chromatogr. A.

[12] Sacher F., Raue B., Klinger J., Brauch H.J. (1999). Simultaneous determination of Cr(III) and Cr(VI) in ground and drinking waters by IC-ICP-MS. Int. J. Environ. Anal. Chem..

[13] Moghadam M.R., Dadfarnia S., Shabani A.M.H. (2011). Speciation and determination of ultra trace amounts of chromium by solidified floating organic drop micro-extraction (SFODME) and graphite furnace atomic absorption spectrometry. J. Hazard. Mater..

[14] Sari T.K., Takahashi F., Jin J., Zein R., Munaf E. (2018). Electrochemical determination of chromium(VI) in river water with gold nanoparticles-graphene nanocomposites modified electrodes. Anal. Sci..

[15] Jin H.G., Zong W.B., Yuan L., Zhang X.B. (2018). Nanoscale zeolitic imidazole framework-90: Selective, sensitive and dual-excitation ratiometric fluorescent detection of hazardous Cr(VI) anions in aqueous media. New J. Chem..

[16] Liu C., Yan B. (2015). Luminescent zinc metal-organic framework (ZIF-90) for sensing metal ions, anions and small molecules. Photochem. Photobiol. Sci..

[17] Li D., Jing P., Sun L., An Y., Shan X., Lu X., Zhou D., Han D., Shen D., Zhai Y. (2018). Near-infrared excitation/emission and multiphoton-induced fluorescence of carbon dots. Adv. Mater..

[18] Batool M., Junaid H.M., Tabassum S., Kanwal F., Abid K., Fatima Z., Shah A.T. (2022). Metal ion detection by carbon dots—A review. Crit. Rev. Anal. Chem..

[19] Ming F., Hou J., Hou C., Yang M., Wang X., Li J., Huo D., He Q. (2019). One-step synthesized fluorescent nitrogen doped carbon dots from thymidine for Cr (VI) detection in water. Spectrochim. Acta A Mol. Biomol. Spectrosc..

[20] Wang H.T., Liu S., Xie Y.S., Bi J.R., Li Y., Song Y.K., Cheng S.S., Li D.M., Tan M.Q. (2018). Facile one-step synthesis of highly luminescent N-doped carbon dots as an efficient fluorescent probe for chromium(vi) detection based on the inner filter effect. New J. Chem..

[21] Zhang Y., Fang X., Zhao H., Li Z. (2018). A highly sensitive and selective detection of Cr(VI) and ascorbic acid based on nitrogen-doped carbon dots. Talanta.

[22] Furukawa H., Cordova K.E., O’Keeffe M., Yaghi O.M. (2013). The chemistry and applications of metal-organic frameworks. Science.

[23] Jones C.G., Stavila V., Conroy M.A., Feng P., Slaughter B.V., Ashley C.E., Allendorf M.D. (2016). Versatile synthesis and fluorescent labeling of ZIF-90 nanoparticles for biomedical applications. ACS Appl. Mater. Interfaces.

[24] Saber-Samandari S., Saber-Samandari S. (2017). Biocompatible nanocomposite scaffolds based on copolymer-grafted chitosan for bone tissue engineering with drug delivery capability. Mater. Sci. Eng. C Mater. Biol. Appl..

[25] Khan W.U., Wang D., Zhang W., Tang Z., Ma X., Ding X., Du S., Wang Y. (2017). High quantum yield green-emitting carbon dots for Fe(III) detection, biocompatible fluorescent ink and cellular imaging. Sci. Rep..

[26] Amjadi M., Abolghasemi-Fakhri Z., Hallaj T. (2015). Carbon dots-silver nanoparticles fluorescence resonance energy transfer system as a novel turn-on fluorescent probe for selective determination of cysteine. J. Photochem. Photobiol. A.

[27] Wang C.I., Wu W.C., Periasamy A.P., Chang H.T. (2014). Electrochemical synthesis of photoluminescent carbon nanodots from glycine for highly sensitive detection of hemoglobin. Green Chem..

[28] Li X., Wang H., Shimizu Y., Pyatenko A., Kawaguchi K., Koshizaki N. (2011). Preparation of carbon quantum dots with tunable photoluminescence by rapid laser passivation in ordinary organic solvents. Chem. Commun..

[29] Tang L., Ji R., Cao X., Lin J., Jiang H., Li X., Teng K.S., Luk C.M., Zeng S., Hao J. (2012). Deep ultraviolet photoluminescence of water-soluble self-passivated graphene quantum dots. ACS Nano.

[30] Manioudakis J., Victoria F., Thompson C.A., Brown L., Movsum M., Lucifero R., Naccache R. (2019). Effects of nitrogen-doping on the photophysical properties of carbon dots. J. Mater. Chem. C.

[31] Atchudan R., Edison T., Mani S., Perumal S., Vinodh R., Thirunavukkarasu S., Lee Y.R. (2020). Facile synthesis of a novel nitrogen-doped carbon dot adorned zinc oxide composite for photodegradation of methylene blue. Dalton Trans..

[32] Tammina S.K., Wan Y., Li Y., Yang Y. (2020). Synthesis of N, Zn-doped carbon dots for the detection of Fe^3+^ ions and bactericidal activity against *Escherichia coli* and *Staphylococcus aureus*. J. Photochem. Photobiol. B.

[33] Das P., Ganguly S., Bose M., Mondal S., Choudhary S., Gangopadhyay S., Das A.K., Banerjee S., Das N.C. (2018). Zinc and nitrogen ornamented bluish white luminescent carbon dots for engrossing bacteriostatic activity and Fenton based bio-sensor. Mater. Sci. Eng. C Mater. Biol. Appl..

[34] Adotey E.K., Torkmahalleh M.A., Balanay M.P. (2020). Zinc metal-organic framework with 3-pyridinecarboxaldehyde and trimesic acid as co-ligands for selective detection of Cr (VI) ions in aqueous solution. Methods Appl. Fluoresc..

[35] Chen B., Wang L., Xiao Y., Fronczek F.R., Xue M., Cui Y., Qian G. (2009). A luminescent metal-organic framework with Lewis basic pyridyl sites for the sensing of metal ions. Angew. Chem. Int. Ed..

[36] Hasanzadeh A., Radmanesh F., Hosseini E.S., Hashemzadeh I., Kiani J., Nourizadeh H., Naseri M., Fatahi Y., Chegini F., Madjd Z. (2021). Highly Photoluminescent nitrogen- and zinc-doped carbon dots for efficient delivery of CRISPR/Cas9 and mRNA. Bioconjug. Chem..

[37] Tammina S.K., Yang D., Li X., Koppala S., Yang Y. (2019). High photoluminescent nitrogen and zinc doped carbon dots for sensing Fe^3+^ ions and temperature. Spectrochim. Acta A.

[38] Zhu Y., Lu Y., Shi L., Yang Y. (2020). β-Cyclodextrin functionalized N,Zn codoped carbon dots for specific fluorescence detection of fluoroquinolones in milk samples. Microchem. J..

[39] Porrès L., Holland A., Pålsson L.-O., Monkman A.P., Kemp C., Beeby A. (2006). Absolute measurements of photoluminescence quantum yields of solutions using an integrating sphere. J. Fluoresc..

[40] Li C., Liu W.J., Sun X.B., Pan W., Wang J.P. (2017). Multi sensing functions integrated into one carbon-dot based platform via different types of mechanisms. Sens. Actuators B.

[41] Song S., Liang F., Li M., Du F., Dong W., Gong X., Shuang S., Dong C. (2019). A label-free nano-probe for sequential and quantitative determination of Cr(VI) and ascorbic acid in real samples based on S and N dual-doped carbon dots. Spectrochim. Acta A Mol. Biomol. Spectrosc..

[42] Bai J.L., Ma Y.S., Yuan G.J., Chen X., Mei J., Zhang L., Ren L.L. (2019). Solvent-controlled and solvent-dependent strategies for the synthesis of multicolor carbon dots for pH sensing and cell imaging. J. Mater. Chem. C.

[43] Jose S.P., Mohan S. (2006). FT-IR and FT-Raman investigations of nicotinaldehyde. Spectrochim. Acta A Mol. Biomol. Spectrosc..

[44] Mahalakshmi G., Balachandran V. (2014). FT-IR and FT-Raman spectra, normal coordinate analysis and ab initio computations of trimesic acid. Spectrochim. Acta A Mol. Biomol. Spectrosc..

[45] Thommes M., Kaneko K., Neimark A.V., Olivier J.P., Rodriguez-Reinoso F., Rouquerol J., Sing K.S.W. (2015). Physisorption of gases, with special reference to the evaluation of surface area and pore size distribution (IUPAC Technical Report). Pure Appl. Chem..

[46] Song J.G., Wang X.Z., Chang C.T. (2014). Preparation and Characterization of Graphene Oxide. J. Nanomater..

[47] Siburian R., Sihotang H., Raja S.L., Supeno M., Simanjuntak C. (2018). New route to synthesize of graphene nano sheets. Orient. J. Chem..

[48] Zhang Y., Gao Z., Yang X., Yang G., Chang J., Jiang K. (2019). Highly fluorescent carbon dots as an efficient nanoprobe for detection of clomifene citrate. RSC Adv..

[49] Torkmahalleh M.A., Lin L., Holsen T.M., Rasmussen D.H., Hopke P.K. (2012). The impact of deliquescence and pH on Cr speciation in ambient PM samples. Aerosol Sci. Technol..

[50] Ganya E.S., Soin N., Moloi S.J., McLaughlin J.A., Pong W.F., Ray S.C. (2020). Polyacrylate grafted graphene oxide nanocomposites for biomedical applications. J. Appl. Phys..

[51] Astafiev A.A., Shakhov A.M., Tskhovrebov A.G., Shatov A., Gulin A., Shepel D., Nadtochenko V.A. (2022). Nitrogen-doped carbon nanodots produced by femtosecond laser synthesis for effective fluorophores. ACS Omega.

[52] Biesinger M.C., Brown C., Mycroft J.R., Davidson R.D., McIntyre N.S. (2004). X-ray photoelectron spectroscopy studies of chromium compounds. Surf. Interface Anal..

[53] Wang Q.Q., Zhang S.D., Yu Y.Z., Dai B. (2020). High-performance of plasma-enhanced Zn/MCM-41 catalyst for acetylene hydration. Catal. Commun..

[54] Huang X.F., Xie Z.W., Li K.S., Chen Q., Chen Y.J., Gong F. (2020). Thermal stability of CrWN glass molding coatings after vacuum annealing. Coatings.

[55] López J.C., Alkorta I., Macario A., Blanco S. (2022). Characterizing the n→π* interaction of pyridine with small ketones: A rotational study of pyridine⋯acetone and pyridine⋯2-butanone. Phys. Chem. Chem. Phys..

[56] López J.C., Macario A., Maris A., Alkorta I., Blanco S. (2021). How aromatic fluorination exchanges the interaction role of pyridine with carbonyl compounds: The formaldehyde adduct. Chem. Eur. J..

[57] Wang J.L., Wu Z.L., Chen S.H., Yuan R., Dong L. (2019). A novel multifunctional fluorescent sensor based on N/S co-doped carbon dots for detecting Cr (VI) and toluene. Microchem. J..

[58] Bandara P.C., Pena-Bahamonde J., Rodrigues D.F. (2020). Redox mechanisms of conversion of Cr(VI) to Cr(III) by graphene oxide-polymer composite. Sci. Rep..

[59] Bakhtiari N., Azizian S. (2015). Adsorption of copper ion from aqueous solution by nanoporous MOF-5: A kinetic and equilibrium study. J. Mol. Liq..

[60] Noraee Z., Jafari A., Ghaderpoori M., Kamarehie B., Ghaderpoury A. (2019). Use of metal-organic framework to remove chromium (VI) from aqueous solutions. J. Environ. Health Sci. Eng..

[61] Maleki A., Hayati B., Naghizadeh M., Joo S.W. (2015). Adsorption of hexavalent chromium by metal organic frameworks from aqueous solution. J. Ind. Eng. Chem..

[62] Kaur H., Sinha S., Krishnan V., Koner R.R. (2020). Photocatalytic reduction and recognition of Cr(VI): New Zn(II)-based metal-organic framework as catalytic surface. Ind. Eng. Chem. Res..

[63] Lin L., Rong M., Lu S., Song X., Zhong Y., Yan J., Wang Y., Chen X. (2015). A facile synthesis of highly luminescent nitrogen-doped graphene quantum dots for the detection of 2,4,6-trinitrophenol in aqueous solution. Nanoscale.

[64] Liu Y.S., Qu B.H., Li Z.J., Yan R., Li P., Zhang Z.S., Yin Y.D., Jing L.Q. (2019). Improved fluorescence test of chromium (VI) in aqueous solution with g-C_3_N_4_ nanosheet and mechanisms. Mater. Res. Bull..

