# Supplementary material for: N,Zn-Doped Fluorescent Sensor Based on Carbon Dots for the Subnanomolar Detection of Soluble Cr(VI) Ions
Source: Sensors (Basel). 2023 Feb 2;23(3):1632. doi: 10.3390/s23031632 (PMC9919354; doi:10.3390/s23031632)
Supplement: Supplementary file 1 [file sensors-2113198-supplementary.pdf › sensors-2113198-supplementary.pdf]

# N,Zn-Doped Fluorescent Sensor Based on Carbon Dots for the Subnanomolar Detection of Soluble Cr(VI) Ions

**Enoch Kwasi Adotey [1], Mehdi Amouei Torkmahalleh [2], Philip K. Hopke [3] and Mannix P. Balanay [4,*]**

[1] Department of Chemical and Material Engineering, Nazarbayev University, Astana 010000, Kazakhstan; enoch.adotey@nu.edu.kz
[2] Division of Environmental and Occupational Health Sciences, School of Public Health, University of Illinois at Chicago, Chicago, IL 60612, USA; mehdiat@uic.edu
[3] Department of Public Health Sciences, University of Rochester School of Medicine and Dentistry, Rochester, NY 14642, USA; phopke@clarkson.edu
[4] Department of Chemistry, Nazarbayev University, Astana 010000, Kazakhstan
* Correspondence: mannix.balanay@nu.edu.kz; Tel.: +7-7172-694657

## Supplementary Information

### Instrumentation

An infrared spectroscopy system for routine analytical purposes was performed using the Nicolet iS10 FT-IR spectrometer. Fluorescence experiments were performed using a Cary Eclipse fluorescence spectrophotometer (Agilent Technologies). The width of the excitation and emission slits was set to 20.0 nm for each of the fluorescence experiments. X-ray diffraction (XRD) patterns were performed using a Rigaku SmartLab® X-ray diffraction system at an angular range of 5-75° and a sampling rate of 5° $min^{-1}$. Scanning electron microscopy images (SEM) were acquired using a FeSEM Auriga (Crossbeam 540) scanning electron microscope. The microscope configuration of the FeSEM allows us to perform energy dispersive spectroscopy (EDS), imaging, and analysis of the GEMINI column associated with the particles. The nitrogen porosimeter (automated gas sorption analyzer) was used to determine the porosity and surface area of the particles. Thermogravimetric analyzes (TGA) were performed using the Simultaneous Thermal Analyzer (STA) 6000 under the following conditions: Air heating rate of 5°/min, air flow pressure of 20 ml/min, and temperature range of 30-600 °C. The pH of the solutions was measured using the HI 2550 pH/ORP & EC/TDS/NaCl Benchtop Meter (Hanna Instruments, Denmark) and the zeta potential was measured using the ZN3600 Zetasizer (Malvern Panalytical Ltd.) at room temperature. The Nexsa X-ray photoelectron spectrometer (XPS) (Thermo Scientific) was used to study the surface chemistry, elemental composition, chemical state, and electronic state of the elements in the nanomaterial. Transmission electron microscope JEOL JEM - 1400 Plus was used to obtain high-quality images of the size and crystallography of the synthesized nanomaterial. The fluorescence decay time and quantum yield were determined using Edinburgh Instruments FLS1000 photofluorescence spectrometer (Edinburgh Instruments Ltd.). The quantum yield of the N,Zn-doped CDs was calculated using the absolute method, which is defined by the sum of all emitted photons divided by the sum of all absorbed photons [1].

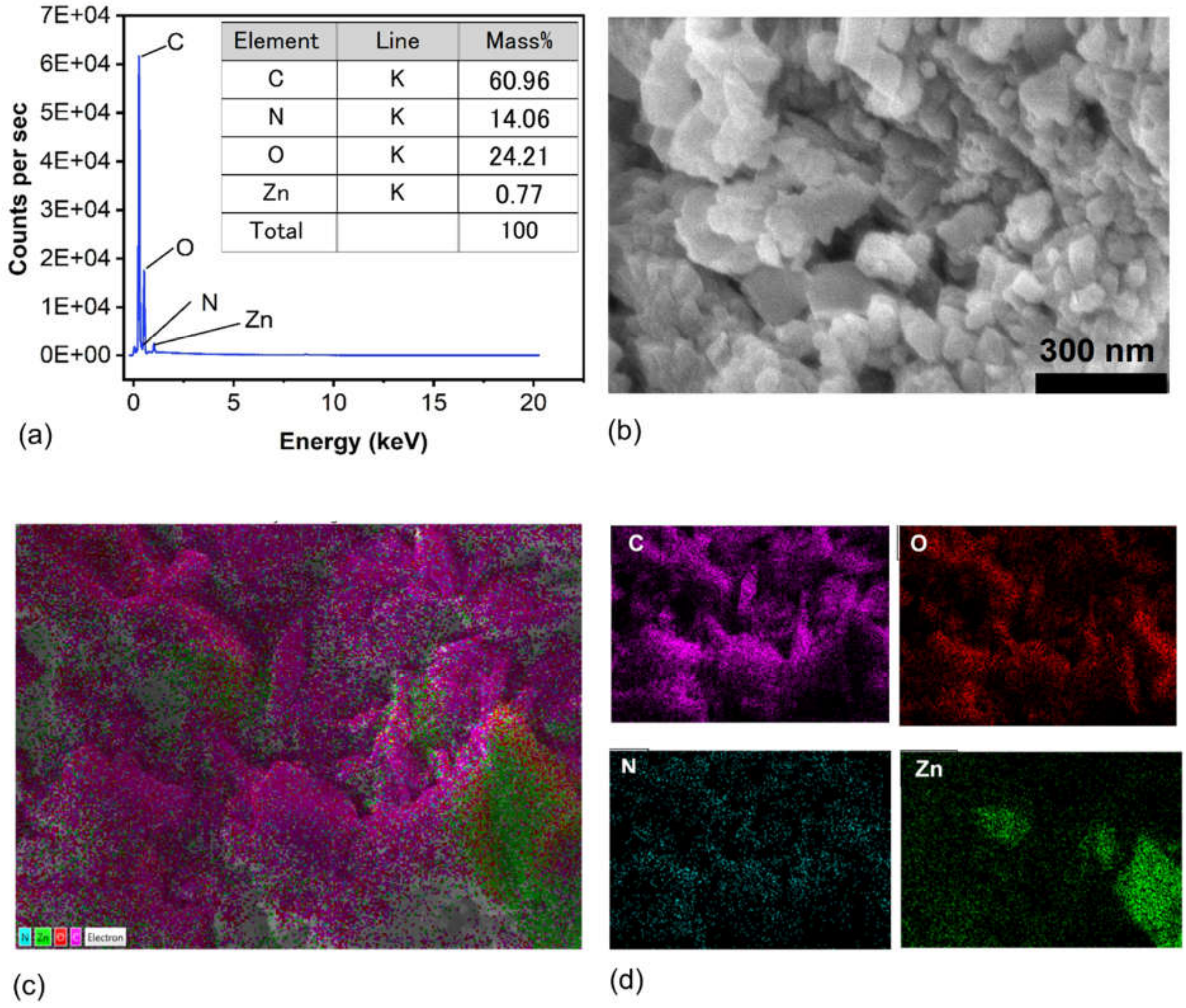


**Figure S1.** (**a**) EDS composition, (**b**) SEM image, and (**c**) to (**d**) elemental mapping of the as-synthesized particles.

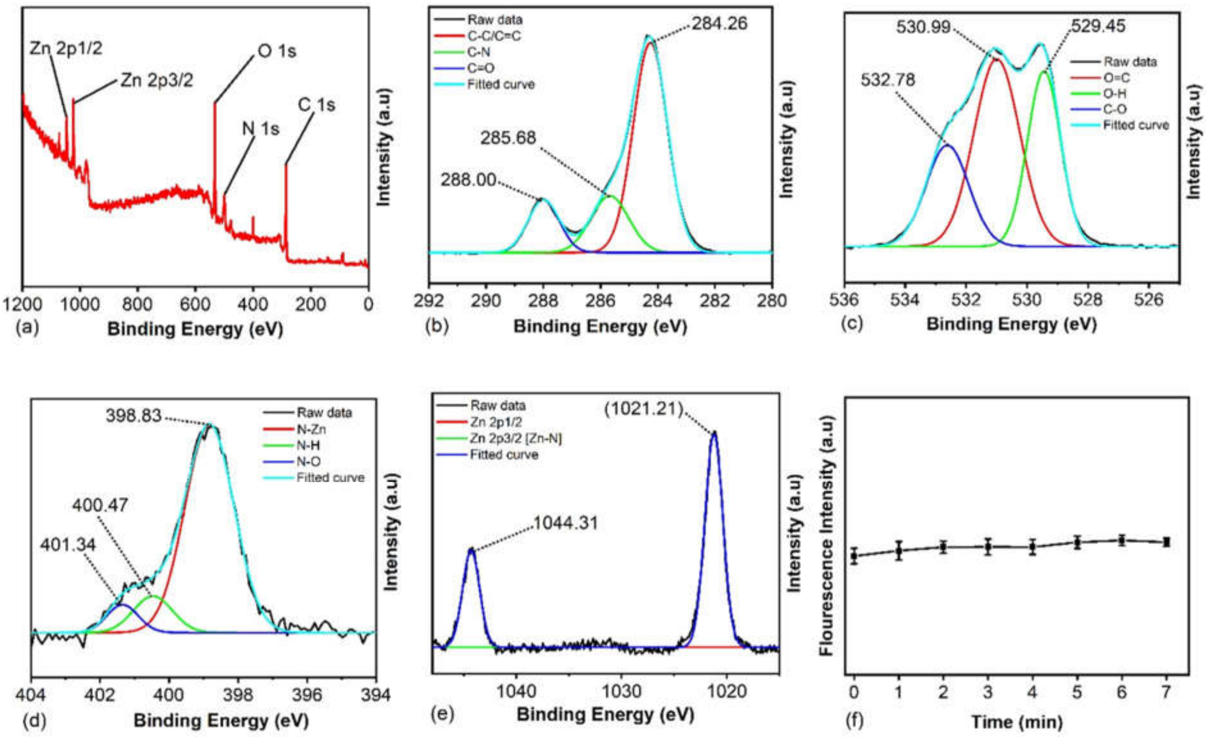


**Figure S2.** XPS spectra of N, Zn-CDs (**a**) survey spectrum, (**b**) carbon deconvoluted peaks (C1s), (**c**) oxygen deconvoluted peaks (O1s), (**d**) nitrogen deconvoluted peaks (N1s), and (**e**) zinc deconvoluted peaks (Zn2p). (**f**) Comparison of the quenching stability of the N,Zn-CDs suspension over a few minutes.

**Table S1.** The fluorescence decay of the N,Zn-CD + incremental volumes addition of 5 ppb Cr(VI) system fitted to a three-exponential function.

| Systems | $\tau_1$ | $\tau_2$ | Avg. |
|---|---|---|---|
| N,Zn-CDs | 2.75 | 2.38 | 2.57 |
| N,Zn-CDs + 40 µL | 2.64 | 2.43 | 2.53 |
| N,Zn-CDs + 80 µL | 2.99 | 2.54 | 2.77 |
| N,Zn-CDs + 120 µL | 2.83 | 2.51 | 2.67 |
| N,Zn-CDs + 160 µL | 2.83 | 2.58 | 2.71 |
| N,Zn-CDs + 240 µL | 2.73 | 2.45 | 2.59 |

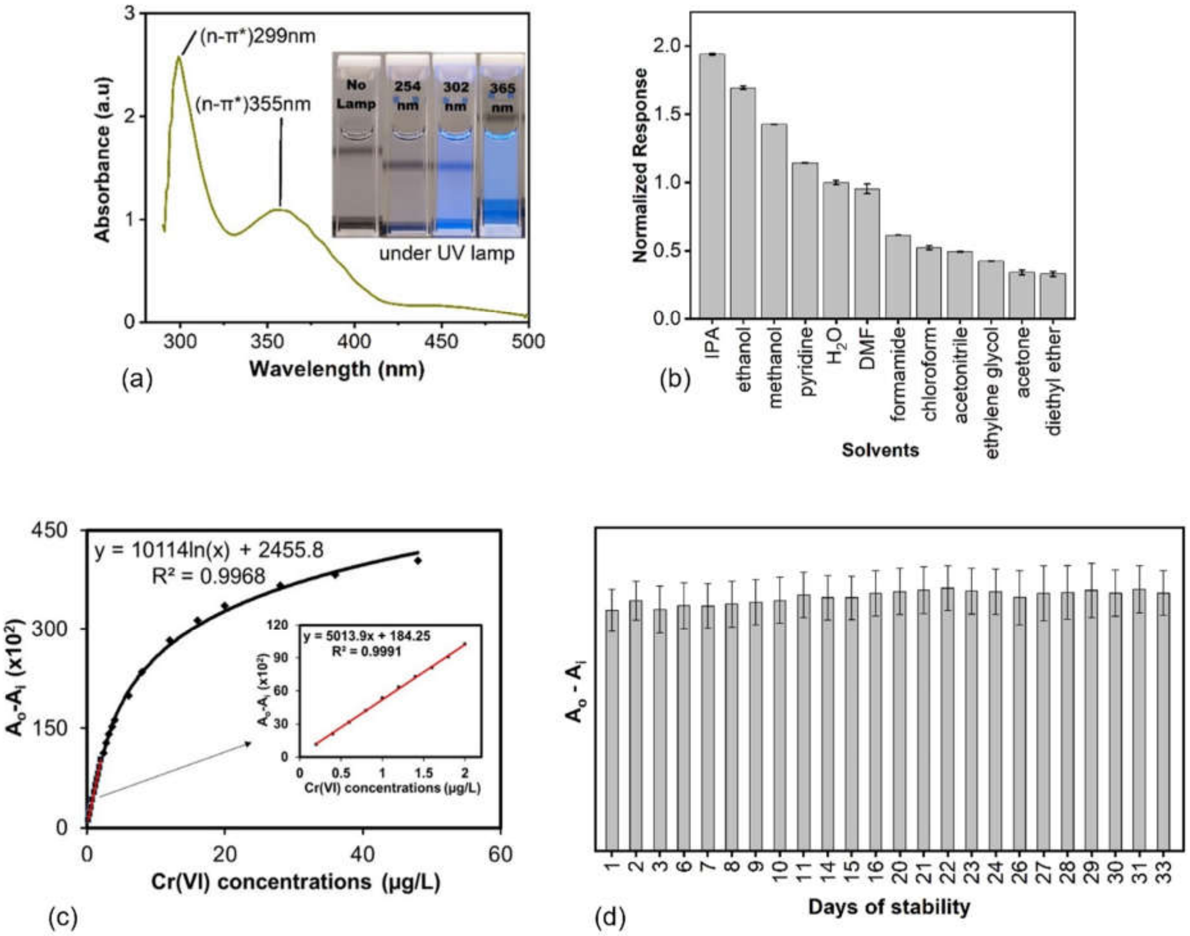


**Figure S3.** (**a**) UV-Vis absorption spectrum of the N,Zn-CDs + Cr system; the inset shows the solution under the UV lamp at 254, 302, and 365 nm, (**b**) fluorescence response of the as-synthesized nanomaterial to various organic solvents, (**c**) a plot of low and high Cr(VI) concentrations when added to the N,Zn-CDs suspension (inset: the calibration curve showing the linear trend of Cr(VI) at very low concentrations), and (**d**) Influence on the stability of fluorescence intensity of N,Zn-CDs in water after several days.

**Variations of the precursors and some characterizations**

The ratios of zinc nitrate hexahydrate and 4-pyridinecarboxyaldehyde were varied to determine the influence of the two precursors on the detection of Cr(VI). Meanwhile, control CDs ((a) without Zn doping and (b) without N doping) were synthesized using a similar microwave technique. In the absence of Zn (denoted here as CD + N) in the starting material, a dark brown solution was obtained, but no precipitate was collected. Similarly, no precipitate was present when the main nitrogen source (4-pyridinecarboxyaldehyde) was not used as part of the starting material (denoted as CD + Zn). The UV-Vis images at 365 nm of the control CDs and other variations are shown in Figure S4. It can be observed that the suspensions show a blue coloration under the UV lamp, except for the sample CD +Zn. Therefore, we assume that the samples that showed blue coloration have fluorescent properties.

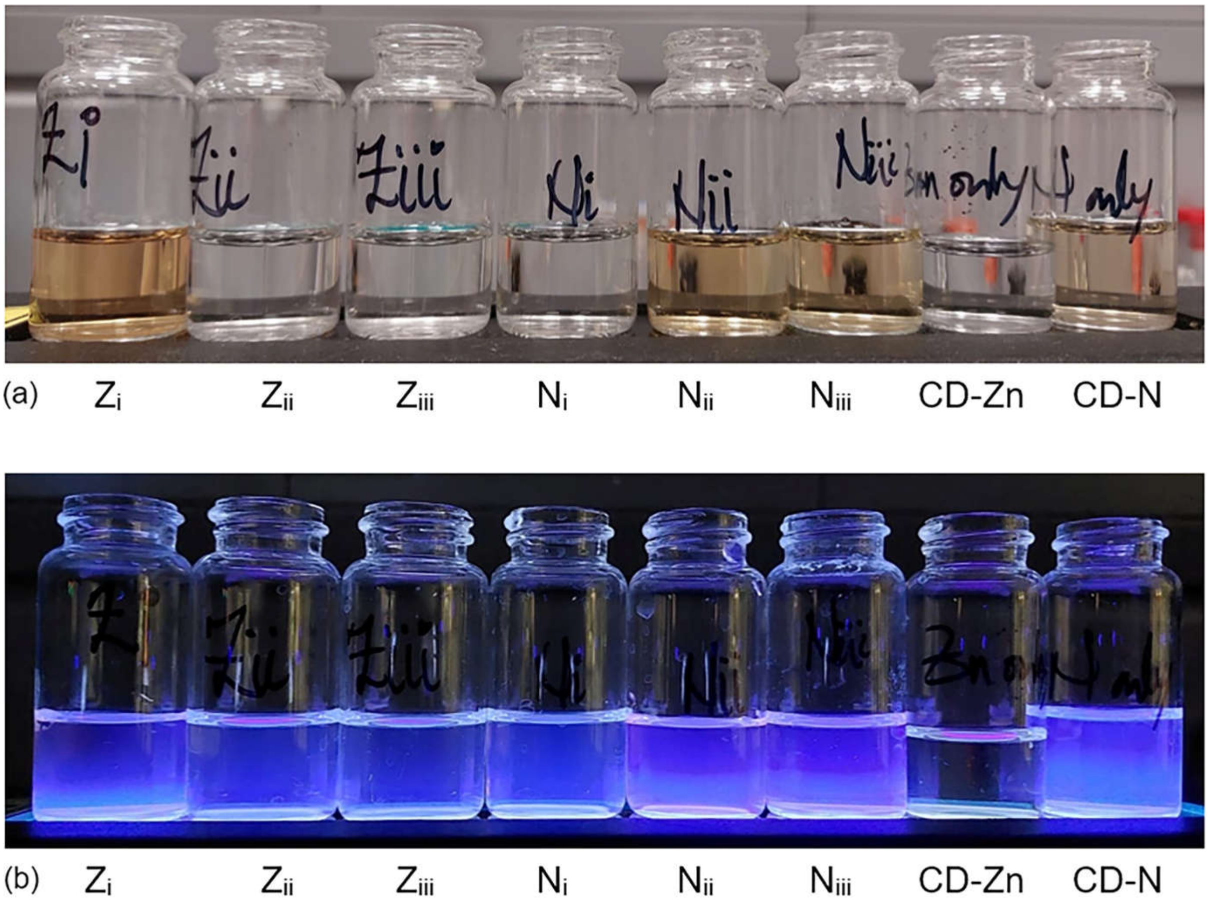


**Figure S4.** CD suspensions (**a**) at room temperature and (**b**) observed under a UV lamp at 365 nm.

Table S2 shows the variations of the starting materials (N and Zn) used in this study. The synthesis procedure is the same as described in the Methodology section of the manuscript. Briefly, the temperature was slowly increased for 10 minutes until it reached 200°C and then maintained at a pressure of < 200 psi for 30 minutes. Precipitates formed only in samples containing ID; $N_{ii}$, $N_{iii}$, $Z_{ii}$ and $Z_{iii}$. The remaining samples (Ni, Zi, CD + Zn, CD + N) were in liquid form only. Since the focus of this study is on the use of the precipitates from the resulting as-synthesized samples, we present here only the characterization of the samples containing ID; $N_{ii}$, $N_{iii}$, $Z_{ii}$, and $Z_{iii}$, which could potentially be used in the future. Based on the lowest LOD in Table S2, the N,Zn-CDs were selected and mainly used in this study.

**Table S2.** Variations in the use of starting materials during synthesis and its LOD.

| Sample ID | CA (mg) | 4-Py (µL) | Zn (mg) | Comment | LOD (nmol/L) |
|---|---|---|---|---|---|
| $Z_i$ | 420.2 | 200 | 117.8 | Variation of the concentration of Zn | - |
| N,Zn-CDs | | | 235.5 | | **0.47** |
| $Z_{ii}$ | | | 471.2 | | 3.35 |
| $Z_{iii}$ | | | 706.5 | | 1.08 |
| $N_i$ | 420.2 | 100 | 235.5 | Variation of the concentration of nitrogen | - |
| $N_{ii}$ | | 400 | | | 3.14 |
| $N_{iii}$ | | 600 | | | 1.59 |

Note: CA: citric acid monohydrate; 4-Py: 4-pyridinecarboxyaldehyde; Zn: Zn(II) nitrate hexahydrate.

The FTIR spectra of the control samples are shown in Figure S5. A well-defined peak at 3435 $cm^{-1}$ was observed in all samples, which was attributed to the stretching vibration of the -OH group in the structure of the CDs [2-7]. However, samples $Z_{iii}$ showed lower stretching, indicating that a smaller amount of -OH groups is present. Weak peaks at 3104 and 3040 $cm^{-1}$ can be associated with the vibrational bands of =CH and -CH, respectively [4, 5, 7]. The -CN peaks [2, 3, 5] at 2354 $cm^{-1}$ can be attributed to the stretching vibration of the pyridyl nitrogen present in the sample, and sample $N_{iii}$ showed the broadest peak in this region as it contained the largest amount of 4-pyridinecarboxyaldehyde as starting material. The peaks at 1697, 1077 and 1025 $cm^{-1}$ are associated with the C=O stretching vibrations of the aromatic rings in 4-pyridinecarboxaldehyde and the carboxyl group in citric acid [8]. The trend shows that the higher the amount of 4-pyridinecarboxaldehyde as the starting material, the higher the intensity of such a peak, and so the trend was $N_{iii}$ > $N_{ii}$ > $Z_{iii}$ > $Z_{ii}$. The peak at 1407 $cm_{-1}$ can be attributed to the -CHO out-of-plane deformation and C-N stretching vibration of 4-pyridinecarboxaldehyde [8, 9]. The vibration band at 1615 $cm^{-1}$ is attributed to the asymmetric stretching vibration of the carboxyl group (-COOH) in citric acid [6, 9, 10] and was present only in samples $N_{ii}$ and $N_{iii}$. No visible peak at 1615 $cm^{-1}$ was present in samples $Z_{ii}$ and $Z_{iii}$. A peak at 758 $cm^{-1}$ was seen in each sample due to the in-plane and out-of-plane stretching of the aromatic C-H groups of the benzene rings [9, 10]. Nitrates are associated with peaks at 1380, 839, and 670 $cm^{-1}$, and samples $Z_{ii}$ and $Z_{iii}$ show peaks at 1376 and 675 $cm^{-1}$ due to the antisymmetric deformation of $NO_3^-$ from the zinc nitrate included in the synthesis. Samples $N_{ii}$ and $N_{iii}$, on the other hand, showed possible nitrate groups at 673 $cm^{-1}$.

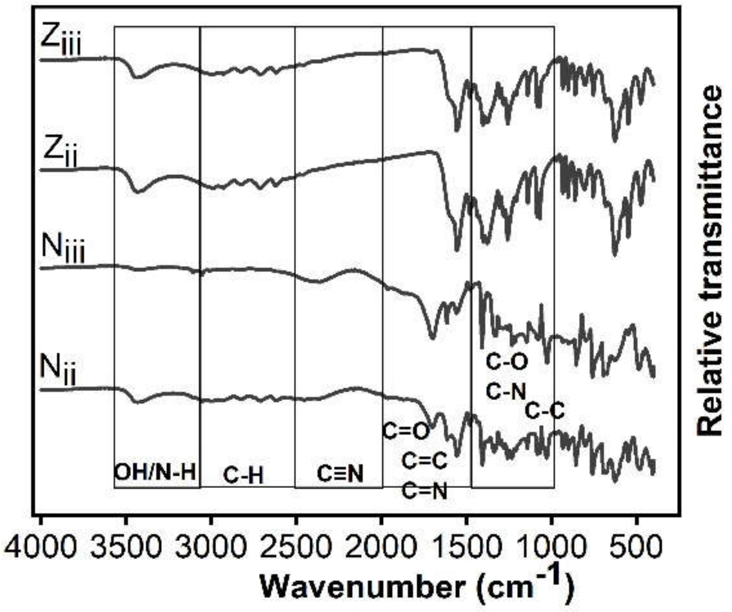


**Figure S5.** FTIR spectra for the variation of the precursors.

The XRD pattern of graphite plotted with the other sample helped assign the peak (Figure S6). The diffraction peak at 26.3° resembled the characteristic peak of graphite ({002} plane, 2θ = 26.5°). Sample $N_{iii}$ with the highest content of 4-pyridinecarboxyaldehyde had the highest intensity at 2θ = 26.3°. The peak at about 2θ = 19.1° was assigned to the phase designation zinc cyanide (301). Sample $Z_{iii}$ with the highest Zn content had the highest intensity at this peak. The diffraction peak at 2θ = 12.2°, thought to be similar to the characteristic peak of graphene oxide (2θ = 10.6°, {001} plane), was predominant in the samples, and again sample $Z_{iii}$ had the most intense peak. Thus, the inclusion of zinc in the starting material tends to influence the formation of this peak.

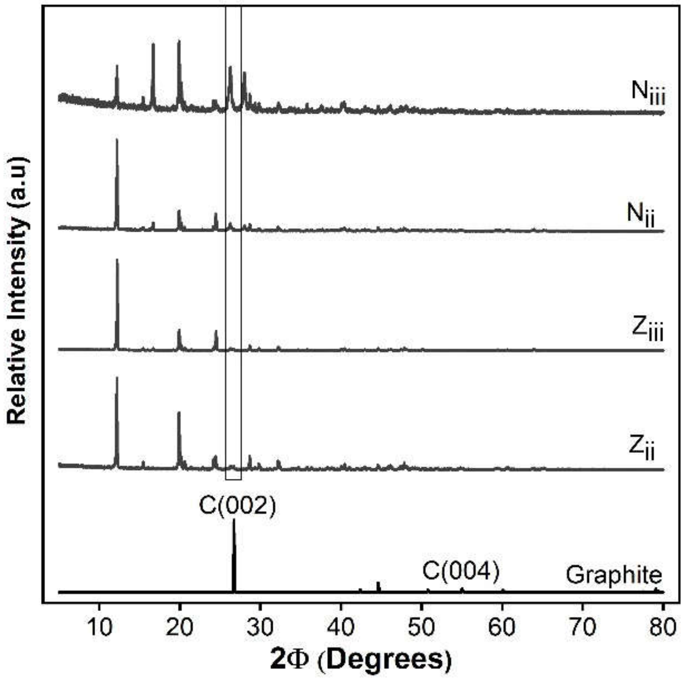


**Figure S6.** XRD diffraction spectra for graphite and the variation of precursors.

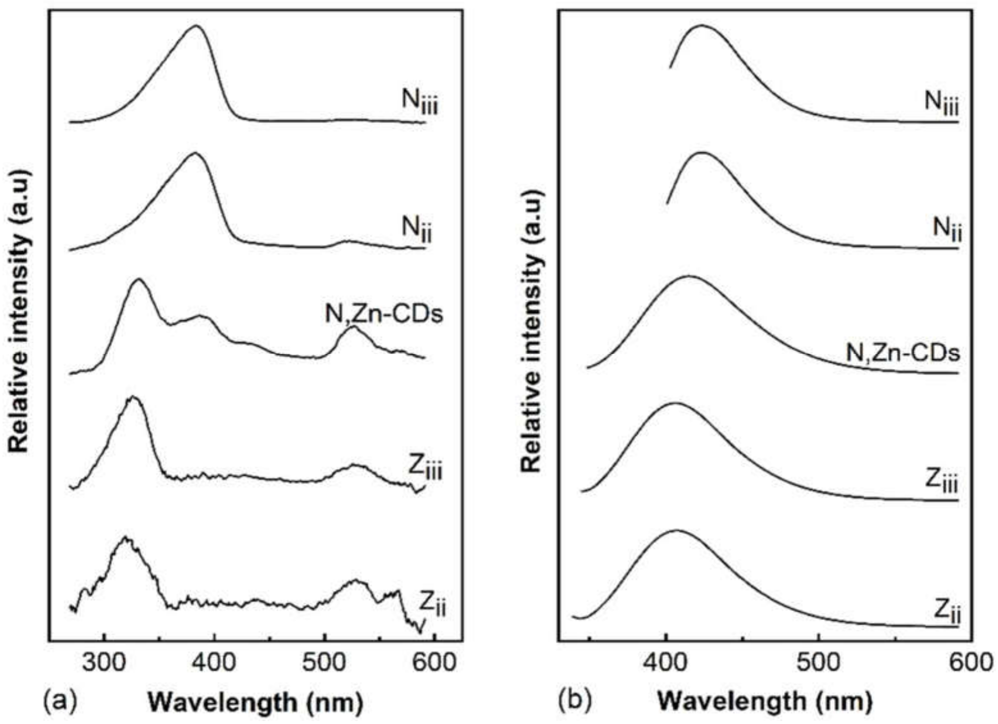


**Figure S7.** (**a**) Excitation and (**b**) emission spectra of the various precursor combinations.

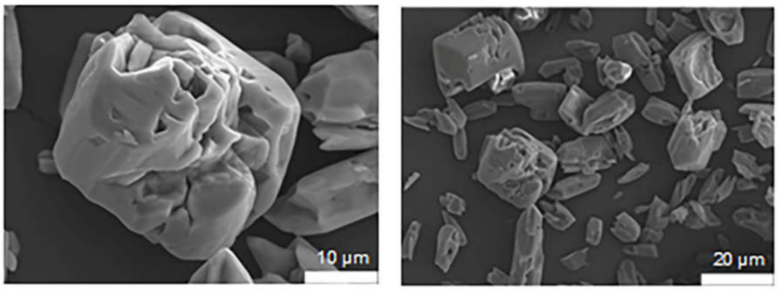


| Element | Mass% | Atom% |
|---|---|---|
| C | 48.50±0.23 | 57.10±0.27 |
| N | 10.17±0.36 | 10.26±0.36 |
| O | 35.50±0.45 | 31.38±0.40 |
| Zn | 5.83±0.49 | 1.26±0.11 |

(a)

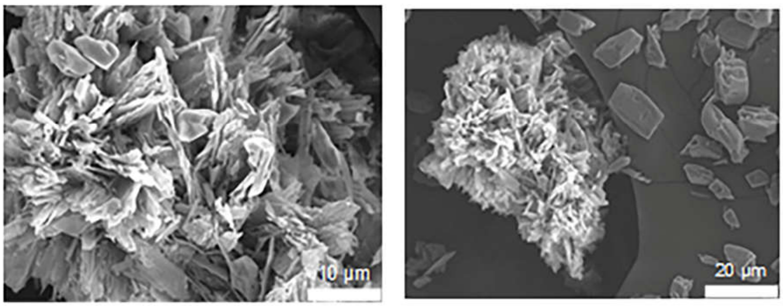


| Element | Mass% | Atom% |
|---|---|---|
| C | 52.10±0.34 | 60.79±0.40 |
| N | 10.19±0.53 | 10.20±0.53 |
| O | 31.65±0.62 | 27.72±0.55 |
| Zn | 6.05±0.70 | 1.30±0.15 |

(b)

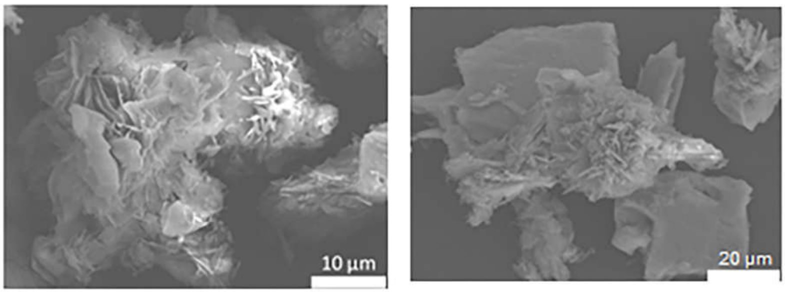


| Element | Mass% | Atom% |
|---|---|---|
| C | 50.50±0.11 | 59.76±0.14 |
| N | 13.14±0.20 | 13.33±0.20 |
| O | 29.55±0.22 | 26.25±0.19 |
| Zn | 1.17±0.12 | 0.25±0.03 |

(c)

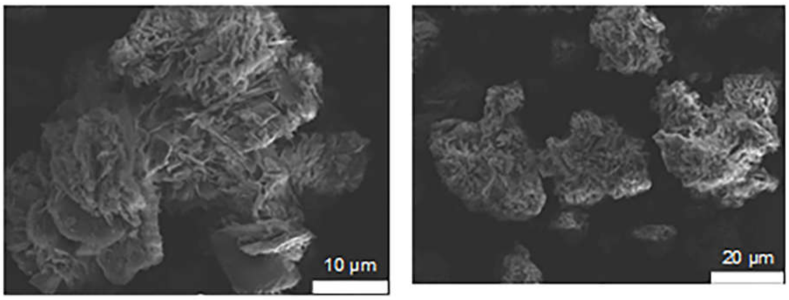


| Element | Mass% | Atom% |
|---|---|---|
| C | 49.99±0.16 | 57.61±0.18 |
| N | 13.77±0.28 | 13.60±0.27 |
| O | 32.31±0.31 | 27.96±0.27 |
| Zn | 3.93±0.28 | 0.83±0.06 |

(d)

**Figure S8.** SEM/EDS analyses for the variation of precursors with ID (**a**) $Z_{ii}$, (**b**) $Z_{iii}$, (**c**) $N_{ii}$, and (**d**) $N_{iii}$.

## References


1. Porrès, L.; Holland, A.; Pålsson, L. O.; Monkman, A. P.; Kemp, C.; Beeby, A., Absolute measurements of photoluminescence quantum yields of solutions using an integrating sphere. *J Fluoresc* **2006,** 16, (2), 267-72.
2. Amjadi, M.; Abolghasemi-Fakhri, Z.; Hallaj, T., Carbon dots-silver nanoparticles fluorescence resonance energy transfer system as a novel turn-on fluorescent probe for selective determination of cysteine. *J Photoch Photobio A* **2015,** 309, 8-14.
3. Li, C.; Liu, W. J.; Sun, X. B.; Pan, W.; Wang, J. P., Multi sensing functions integrated into one carbon-dot based platform via different types of mechanisms. *Sensor Actuat B-Chem* **2017,** 252, 544-553.
4. Ming, F.; Hou, J.; Hou, C.; Yang, M.; Wang, X.; Li, J.; Huo, D.; He, Q., One-step synthesized fluorescent nitrogen doped carbon dots from thymidine for Cr (VI) detection in water. *Spectrochim. Acta, Part A* **2019,** 222, 117165.
5. Song, S.; Liang, F.; Li, M.; Du, F.; Dong, W.; Gong, X.; Shuang, S.; Dong, C., A label-free nano-probe for sequential and quantitative determination of Cr(VI) and ascorbic acid in real samples based on S and N dual-doped carbon dots. *Spectrochim. Acta, Part A* **2019,** 215, 58-68.
6. Wang, H. T.; Liu, S.; Xie, Y. S.; Bi, J. R.; Li, Y.; Song, Y. K.; Cheng, S. S.; Li, D. M.; Tan, M. Q., Facile one-step synthesis of highly luminescent N-doped carbon dots as an efficient fluorescent probe for chromium(vi) detection based on the inner filter effect. *New J Chem* **2018,** 42, (5), 3729-3735.
7. Bai, J. L.; Ma, Y. S.; Yuan, G. J.; Chen, X.; Mei, J.; Zhang, L.; Ren, L. L., Solvent-controlled and solvent-dependent strategies for the synthesis of multicolor carbon dots for pH sensing and cell imaging. *J Mater Chem C* **2019,** 7, (31), 9709-9718.
8. Adotey, E. K.; Amouei Torkmahalleh, M.; Balanay, M. P., Zinc metal-organic framework with 3-pyridinecarboxaldehyde and trimesic acid as co-ligands for selective detection of Cr (VI) ions in aqueous solution. *Methods Appl Fluores* **2020,** 8, (4), 045007.
9. Jose, S. P.; Mohan, S., FT-IR and FT-RAMAN investigations of nicotinaldehyde. *Spectrochim. Acta, Part A* **2006,** 64, (1), 205-9.
10. Mahalakshmi, G.; Balachandran, V., FT-IR and FT-Raman spectra, normal coordinate analysis and ab initio computations of Trimesic acid. *Spectrochim. Acta, Part A* **2014,** 124, 535-47.
